# Minimally Invasive Management of a Hilar Splenic Artery Aneurysm: A Case Report

**DOI:** 10.7759/cureus.93095

**Published:** 2025-09-24

**Authors:** Mehmet Gunay, Suleyman Yilmaz

**Affiliations:** 1 General Surgery, Ministry of Health Beykoz State Hospital, Istanbul, TUR

**Keywords:** case reports, laparoscopy, minimally invasive surgical procedures, splenectomy, splenic artery aneurysm, vascular surgical procedures

## Abstract

Splenic artery aneurysm (SAA) is a rare vascular condition associated with a high risk of rupture, particularly in women of reproductive age. We report the case of a 28-year-old woman with a history of cesarean section and laparoscopic cholecystectomy who presented with persistent, nonspecific abdominal discomfort. Imaging revealed a 2 cm saccular, true aneurysm at the splenic hilum. Following multidisciplinary discussion, endovascular treatment was not considered feasible due to the aneurysm’s hilar location and the associated risk of splenic infarction. Surgical treatment was therefore planned, and a laparoscopic splenectomy was chosen. The procedure was performed using two 10 mm and one 5 mm trocars. Dense hilar fibrosis was encountered intraoperatively; vascular control was carefully established before dissection to reduce the risk of bleeding. The spleen was removed via the previous Pfannenstiel incision without complications. The patient recovered uneventfully and was discharged on postoperative day 3. Histopathological examination confirmed a true, unruptured aneurysm. This case highlights that laparoscopic splenectomy can be a safe and effective treatment option for selected patients with hilar SAA. Although the vascular nature of these lesions may raise concerns regarding intraoperative bleeding, a minimally invasive approach may be feasible when performed by surgeons with sufficient laparoscopic experience in appropriately selected cases.

## Introduction

Splenic artery aneurysms (SAAs), although rare overall, represent the most common form of visceral arterial aneurysm [1]. Rupture can be life-threatening, particularly in women during pregnancy, with reported maternal and fetal mortality rates exceeding 70% [1-3]. As such, treatment is generally recommended regardless of aneurysm size in women of childbearing age, pregnant patients, or those with portal hypertension [2,3].

Endovascular approaches are typically favored due to their minimally invasive nature; however, hilar SAAs present unique challenges. Their close proximity to the splenic parenchyma increases the risk of incomplete exclusion, coil migration, or splenic infarction [4].

Herein, we report the case of a young female patient with a hilar SAA that was not amenable to endovascular treatment. She was successfully managed with laparoscopic splenectomy following a multidisciplinary evaluation.

## Case presentation

A 28-year-old woman presented with intermittent abdominal discomfort. Her surgical history included a laparoscopic cholecystectomy performed two years prior for gallstones and a cesarean section via Pfannenstiel incision. The patient had no known history of chronic illnesses, such as hypertension, diabetes, or connective tissue disorders. Despite these interventions, her symptoms persisted.

**Figure 1:**
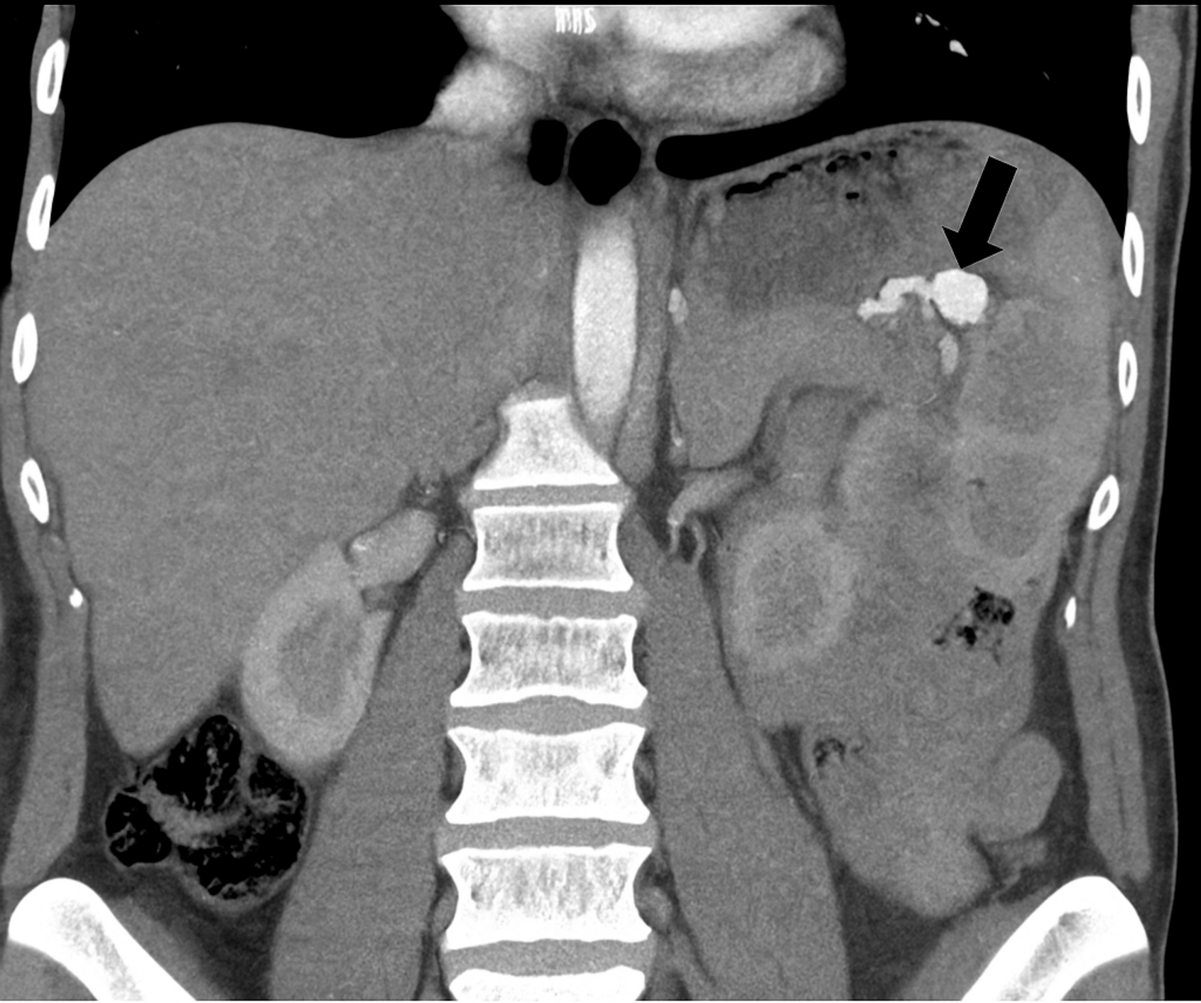
Contrast-enhanced CT angiography showing a 2 cm saccular aneurysm at the splenic hilum.

A contrast-enhanced CT scan revealed a 2 cm saccular SAA located at the splenic hilum (Figure 1). The aneurysm was classified as a true aneurysm, with no signs of rupture.

Following multidisciplinary consultation, including input from interventional radiology, endovascular treatment was deemed unsuitable due to short-neck anatomy, close proximity to the splenic parenchyma, and an increased risk of splenic infarction. The patient was counseled about treatment options, including the potential risks of splenic infarction, abscess formation, and complications from surgical intervention. She elected to proceed with laparoscopic splenectomy.

**Figure 2:**
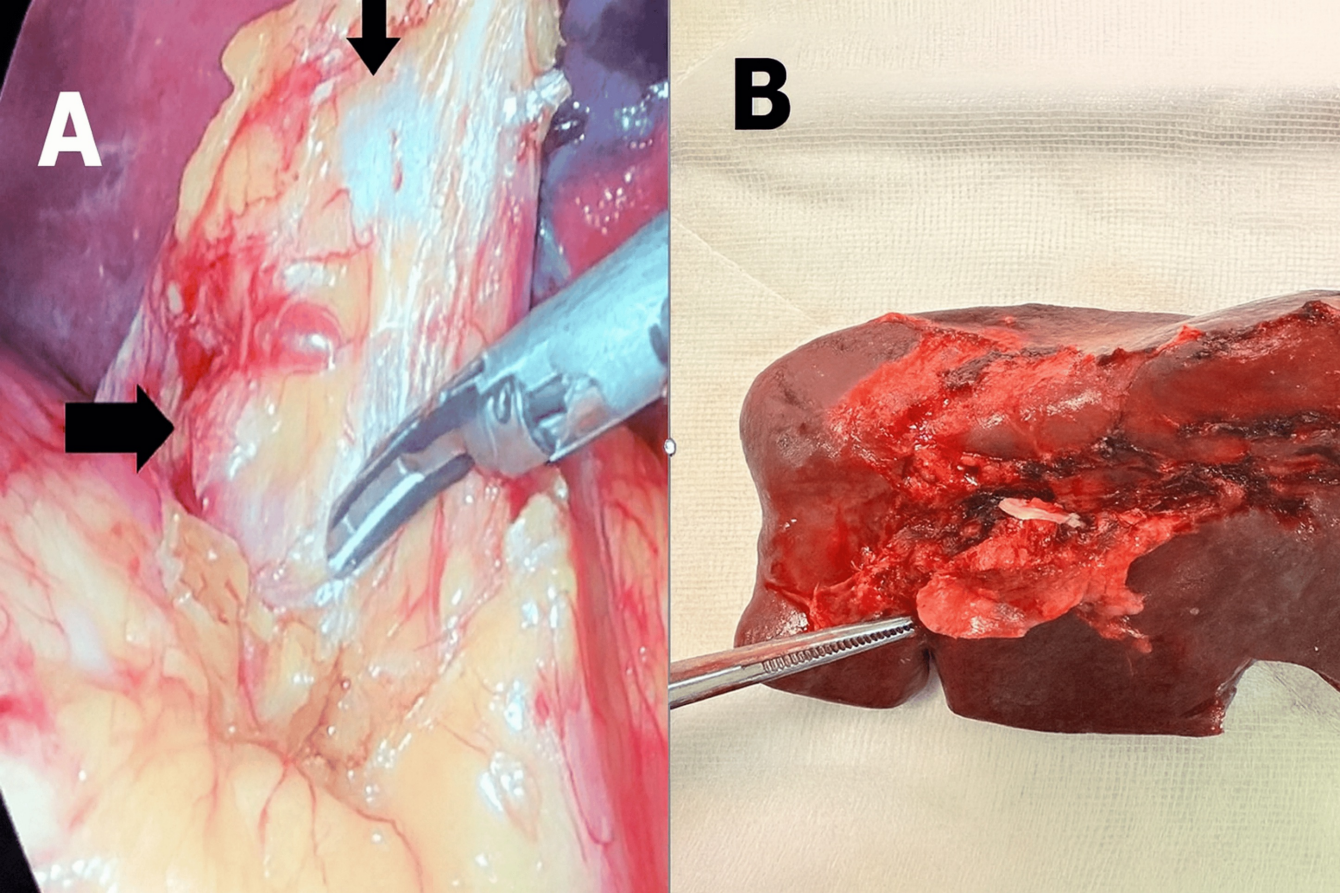
(A) Laparoscopic view of the hilar SAA surrounded by dense adhesions prior to dissection, showing the SAA (upper arrow) and the splenic artery (lateral arrow). (B) Resected spleen specimen showing an unruptured hilar aneurysm distal to the clipped artery, indicated by the tip of the forceps. SAA, splenic artery aneurysm

The procedure was performed using two 10 mm trocars and one 5 mm trocar in the left upper quadrant. The spleen appeared normal in size, and no additional trocars were required. Intraoperatively, dense fibrotic tissue was encountered at the splenic hilum, likely due to the aneurysm (Figure 2A). To minimize rupture risk, proximal vascular control of the splenic artery and vein was achieved before any hilar dissection. The splenectomy proceeded uneventfully, and the spleen was extracted via the prior Pfannenstiel incision (Figure 2B).

Operative time was 90 minutes, with minimal blood loss. The patient recovered without complications and was discharged on postoperative day 3. Histopathological examination confirmed a true, unruptured hilar SAA.

## Discussion

SAAs are often asymptomatic and are typically discovered incidentally. However, their risk of rupture increases significantly during pregnancy, particularly in the third trimester, with maternal and fetal mortality rates reported to exceed 70% [2,3]. In our case, persistent abdominal discomfort following prior cholecystectomy prompted further imaging, which led to the incidental diagnosis of a hilar SAA.

Hilar SAAs present distinct therapeutic challenges due to their proximity to the splenic parenchyma and their short-necked vascular anatomy. These characteristics complicate endovascular access and increase the risk of incomplete exclusion or unintended splenic infarction [4,5]. Although spleen-preserving endovascular techniques are often the preferred approach in younger patients, multidisciplinary evaluation, including consultation with interventional radiology, concluded that the aneurysm’s location and configuration rendered this option unfeasible in our patient.

While endovascular exclusion is generally well tolerated and has become widely accepted, splenic infarction may still occur in up to 40% of patients, particularly when embolization involves the distal third of the artery or the splenic hilum [4]. Large, painful infarcts can necessitate extended hospitalization for pain control, and in some cases, may require splenectomy or percutaneous drainage if complicated by abscess or unrelieved symptoms [4,6].

Preservation of the spleen is preferred unless the SAA is located deep within the hilum. In such cases, endovascular management becomes more technically demanding and raises the risk of incomplete exclusion or nontarget embolization [7]. Recent case series have shown that laparoscopic splenectomy, occasionally accompanied by distal pancreatectomy, can be performed safely and effectively in cases where endovascular treatment is not viable due to hilar anatomy [8]. These findings support our surgical approach in this case, highlighting laparoscopic splenectomy as a feasible alternative for anatomically complex SAAs.

Although spleen-preserving techniques such as laparoscopic aneurysm resection with vascular reconstruction have been reported, their feasibility is largely determined by aneurysm location and intraoperative accessibility. Veterano et al. described a case during pregnancy where the aneurysm was located away from the hilum, making spleen preservation possible through laparoscopic vascular reconstruction [9]. In contrast, our patient had a deeply hilar aneurysm accompanied by dense perihilar fibrosis, precluding safe reconstruction. Therefore, laparoscopic splenectomy was selected as the most definitive and appropriate treatment. This underscores the need for individualized treatment planning based on detailed anatomical and intraoperative assessments.

The patient was thoroughly counseled regarding both endovascular and surgical options, including the risks of splenic infarction, abscess, and surgical complications. After shared decision-making, she opted for surgery. Intraoperatively, dense hilar fibrosis was encountered, likely due to the chronic nature of the aneurysm. Early vascular control was key in preventing rupture. The laparoscopic procedure was completed successfully without complications.

Recent literature supports the efficacy of laparoscopic splenectomy for SAA. In our case, the operative time was 90 minutes, which compares favorably with reported ranges of 80-150 minutes for laparoscopic splenectomy in SAA cases. The patient was discharged on postoperative day 3, consistent with the mean hospital stay of three days reported in contemporary series [8,10]. Blood loss was minimal, aligning with reported values of less than 100-150 mL in laparoscopic approaches [8,10].

Minimally invasive splenectomy, when performed in appropriately selected patients by laparoscopic surgeons, may offer lower morbidity, faster recovery, and improved cosmetic outcomes compared to open surgery, even in vascular pathologies like SAA. Importantly, the patient received preoperative vaccination against encapsulated organisms and had an uneventful postoperative course. At six-month follow-up, CT imaging showed no residual vascular abnormalities or evidence of recanalization, confirming the definitive nature of surgical treatment.

## Conclusions

This case emphasizes the importance of individualized treatment planning in the management of complex hilar SAAs. When endovascular techniques are not feasible due to anatomical limitations, such as short-necked lesions or proximity to the splenic hilum, laparoscopic splenectomy may serve as a safe and effective alternative. Successful outcomes depend on comprehensive preoperative evaluation, careful patient selection, and meticulous intraoperative vascular control. The surgeon’s proficiency in minimally invasive techniques also plays a critical role. When performed under these conditions, laparoscopic splenectomy can minimize postoperative morbidity and expedite recovery, offering favorable outcomes even in vascular pathologies traditionally managed by open surgery. This case supports the growing evidence that minimally invasive surgery should not be overlooked as a viable first-line option in appropriately selected patients with anatomically challenging SAAs.
